# Acute seizure therapies in people with epilepsy: Fact or fiction? A U.S. Perspective

**DOI:** 10.1016/j.ebr.2023.100612

**Published:** 2023-07-08

**Authors:** William O. Tatum, Tracy Glauser, Jurriaan M. Peters, Amit Verma, Sarah Weatherspoon, Selim Benbadis, Danielle A. Becker, Vinay Puri, Michael Smith, Sunita N. Misra, Adrian L. Rabinowicz, Enrique Carrazana

**Affiliations:** a Mayo Clinic, 4500 San Pablo Rd., Jacksonville, FL 32224-1865, USA; b Comprehensive Epilepsy Center, Cincinnati Children’s Hospital, 3333 Burnet Ave., Cincinnati, OH 45229, USA; c Boston Children’s Hospital, Harvard Medical School, 300 Longwood Ave, Boston, MA 02115, USA; d Stanley H. Appel Department of Neurology, Houston Methodist Hospital, 6560 Fannin St., Ste 802, Houston, TX 77030, USA; e Le Bonheur Children's Hospital, University of Tennessee Health Science Center, 848 Adams Ave., Memphis, TN 38103, USA; f Comprehensive Epilepsy Program, University of South Florida & Tampa General Hospital, 2 Tampa General Cir., Tampa, FL 33606, USA; g Department of Neurology, Case Western Reserve University School of Medicine, 10900 Euclid Ave., Cleveland, OH 44106, USA; h Norton Children’s Neuroscience Institute, affiliated with University of Louisville, 411 E. Chestnut St., Suite 645, Louisville, KY 40202, USA; i Department of Neurology, Rush University, 1725 W. Harrison St., Ste 885, Chicago, IL 60612, USA; j Neurelis Inc., 3430 Carmel Mountain Rd., Ste 300, San Diego, CA 92121, USA; k John A. Burns School of Medicine, University of Hawaii, 651 Ilalo St., Honolulu, HI 96813, USA

**Keywords:** Acute repetitive seizures, Benzodiazepines, Rescue therapy, Seizure cluster, Therapeutic misconception

## Abstract

Patients with epilepsy (PWE) may experience seizure emergencies including acute repetitive seizures despite chronic treatment with daily antiseizure medications. Seizures may adversely impact routine daily activities and/or healthcare utilization and may impair the quality of life of patients with epilepsy and their caregivers. Seizures often occur at home, school, or work in a community setting. Appropriate treatment that is readily accessible for patients with seizure urgencies and emergencies is essential outside the hospital setting. When determining the best acute antiseizure therapy for PWE, clinicians need to consider all of the available rescue medications and their routes of administration including the safety and efficacy profiles. Benzodiazepines are a standard of care as a rescue therapy, yet there are several misconceptions about their use and safety. Reevaluating potential misconceptions and formulating best practices are necessary to maximize usage for each available option of acute therapy. We examine common beliefs associated with traditional use of acute seizure therapies to refute or support them based on the current level of evidence in the published literature.

## Highlights

• Untreated seizure emergencies increase the risk of negative outcomes.
• Misconceptions are embedded in traditional use of acute antiseizure medications.
• Potential misconceptions should be reexamined based on the most current evidence.
• Acute seizure therapies have differential benefits and limitations for patients.
• Rapid, appropriate treatment for seizure emergencies is clinically important.

## Introduction

For patients with epilepsy (PWE), seizure urgencies and emergencies include acute repetitive seizures (also known as seizure clusters, which are intermittent increases in seizures that may occur despite treatment with daily antiseizure medication [1]). If left untreated, acute repetitive seizures increase the risk to patients and often lead to negative outcomes. Benzodiazepines are the cornerstone of acute rescue therapy for PWE [2]. Formulations of acute rescue therapies that do not involve intravenous (IV) administration (e.g., intranasal route) are required for rapid treatment of PWE outside the hospital setting in an effort to gain immediate seizure control [3], [4].

In 1997, the United States Food and Drug Administration (FDA) initially approved rectal administration of diazepam for treatment of seizure clusters [5]. This formulation became the primary treatment for PWE that was administered by caregivers outside the hospital setting [5]. However, despite demonstrating efficacy, this route of administration may be associated with limitations to patient or caregiver acceptance resulting in underutilization and undertreatment of PWE [5], [6], [7].

Myths and misbeliefs regarding the limitations of acute rescue therapies may contribute to antiseizure medication (ASM) usage that is not approved by the FDA for this indication [8]. Off-label acute-care use of ASMs may have safety concerns and unknown efficacy as a result, which is concerning for patients with epilepsy. Thus, reevaluating misconceptions associated with acute seizure therapy is needed. Understanding whether these concepts can be supported or refuted should be based on the current clinical evidence for the use of acute seizure therapies.

In this consensus report, we explore misconceptions that exist about the use of acute seizure therapies. Furthermore, we sought to address safety limitations involving both FDA-approved use and off-label use of rescue therapies available for PWE. The objective of this report is to identify potential pitfalls and misconceptions associated with acute seizure therapies and provide clinical pearls about treatment based on evidence obtained from the current treatment landscape.

### Methods

Potential misconceptions about acute seizure therapies were identified from expert consensus comprising authors within the United States (U.S.), focusing on misconceptions and misbeliefs that were selected based on treatment agents for acute seizure urgencies and emergencies. The focus of this paper does not examine misconceptions associated with this type of treatment in the European Union (E.U.) or other areas of the world, which may differ. Viewpoints and perspectives from additional countries and global regions, although valuable for global inclusivity, are not directly aligned with the focus and scope of this U.S.-centered paper. For example, buccal and intranasal midazolam formulations are approved for prolonged acute convulsive seizures in the E.U. [9], [10], but we believe the alignment between this indication and seizure clusters has not been clearly defined for our target audience.

Here, common myths and misconceptions in U.S. practice were stratified by the authors; a narrative review of the literature was performed to obtain a broad perspective. The most relevant ones were selected based on the frequency of occurrence. These misconceptions were then categorized according to patient populations, formulation, route of administration, efficacy, or safety. To dispute or ratify concepts, published literature on treatment of seizure emergencies was accessed using PubMed to support or refute each concept. Using current evidence from selected literature, each concept was designated as either supported or refuted (Table 1). As the intranasal benzodiazepine acute seizure therapies were most recently approved in the U.S., those agents are a main focus here.

**Table 1:** Common misconceptions about intermittent use of acute seizure rescue therapies.

**Misconception?**	**Current Evidence**
Respiratory depression and clinical tolerance over time are uncharacteristic of intermittent intranasal or rectal use of benzodiazepine acute seizure therapies.	In general, respiratory depression and clinical tolerance over time have not been observed with intermittent use of intermittent therapies; a test of the acute seizure therapy should be considered in patients at risk of respiratory depression.

Substantial mucosal nasal irritation is expected with intermittent use of intranasal formulations for patients with seizures.	FDA-approved intranasal formulations are less likely to result in nasal irritation, which is typically mild and transient when it occurs.

Concomitant use of benzodiazepines impacts the safety profile of intermittent use of acute benzodiazepine rescue therapies.	In the phase 3 safety study of diazepam nasal spray, the safety profile in patients taking concomitant benzodiazepines was generally similar to that of other patients in the study.

Acute seizure therapy is intermittently necessary for acute seizure control in children with epilepsy but is not necessary for adult PWE.	Acute seizure therapy is appropriate for both children and adults experiencing seizure emergencies.

Written seizure action plans (SAPs) and acute seizure action plans (ASAPs) only apply to school-aged children with seizures.	All patients with epilepsy should be considered for a customized SAP and/or ASAP.

When administered intranasally as a spray, all benzodiazepine formulations work the same way.	FDA-approved nasal-specific formulations have overcome a number of disadvantages of atomized IV formulations.

Oral benzodiazepines have shortcomings as acute seizure therapy in adults.	Oral medications used off label have shortcomings, including delayed absorption, compared with approved treatments for acute seizure emergencies.

Acute seizure therapy can be safely and easily administered by nonmedical individuals such as care partners and school personnel and in some cases may be self-administered by the patient.	FDA-approved intermittent therapies were developed for use by nonmedical individuals, and self-administration has been documented in some cases.

In patients having a seizure emergency, always wait 5 min or until a second seizure occurs before administering an acute seizure therapy.	Treatment can be initiated once the cluster is recognized; rapid treatment is associated with better outcomes.

Clinical endpoints are the same irrespective of the route of administration for patients requiring an acute seizure rescue therapy.	Effectiveness of a specific formulation varies based on route of administration.

The time to effectiveness of an acute seizure rescue therapy can be determined using pharmacokinetic characteristics.	Time to administration and time to seizure cessation are independent of pharmacokinetic parameters; threshold plasma levels may be of use for estimating therapeutic exposure.

Seasonal allergies and rhinitis do not appear to have an adverse clinical impact on effectiveness of intranasal drug delivery.	Effectiveness of diazepam nasal spray was shown to be maintained in patients with a history or concomitant treatment of seasonal allergies or rhinitis.

FDA, US Food and Drug Administration; IV, intravenous; T_max_, time to maximum plasma concentration.

## Safety

These concepts address respiratory depression, clinical tolerance, and nasal irritation associated with intermittent use of acute seizure therapies.

### Misconception? Respiratory depression and clinical tolerance over time are uncharacteristic of intermittent intranasal or rectal use of benzodiazepine acute seizure therapies

#### Background

Overall, use of benzodiazepines in patients with epilepsy may be associated with concern for cardiorespiratory events such as respiratory arrest and hypoventilation [11]. This is because cardiorespiratory adverse effects have been associated with high-dose IV benzodiazepines [12]. Also, clinical tolerance may occur with chronic use of benzodiazepines by patients with epilepsy [13]. Additionally, a warning labeling exists for benzodiazepines as a class of drugs [14], [15], [16]. This is a requirement by the FDA designed to address potential risks that may occur from concomitant use with central nervous system depressants to avoid misuse or abuse resulting in safety concerns or producing withdrawal reactions [14], [15], [16].

#### Current evidence

In open-label clinical trials for the evaluation of intermittent use of benzodiazepine acute seizure therapies in epilepsy, treatment-related respiratory depression was not reported [17], [18], [19], [20]. In a intranasal midazolam placebo-controlled trial of patients with seizure clusters, during the test-dose phase less than 1% of patients had clinically meaningful treatment-related respiratory depression [21]. In the setting of status epilepticus, the rate of respiratory depression have been shown to be higher in untreated patients than in patients receiving benzodiazepines [22].

Clinical tolerance does not develop during acute intermittent benzodiazepine therapy and was not observed in clinical trials. With rectal diazepam, there was no evidence that higher doses resulted in tolerance nor altered the effectiveness of diazepam [19]. This was demonstrated by no loss of efficacy for seizure reduction in 125 subjects when the patient’s first and last treatment were compared [19]. Continued efficacy without developing tolerance to treatment was shown with repeated intermittent use of intranasal midazolam for seizure clusters in 161 PWE [20]. When the proportion of second-dose use of diazepam via nasal spray was used as a proxy for single-dose effectiveness when treating individual seizure clusters in 79 of 163 PWE, there was a low rate of second-dose use overall (used for 12.6% of seizure clusters) [18]. In subgroup analyses of patients taking concomitant benzodiazepines or receiving diazepam nasal spray more than twice a month, tolerance was not a concern, and there were no clinically relevant differences in effectiveness [18].

#### Conclusion

Respiratory depression and clinical tolerance over time are NOT characteristic of benzodiazepine acute seizure therapies.

### Misconception? Substantial mucosal nasal irritation is expected with intermittent use of intranasal formulations for patients with seizures

#### Background

Nasal discomfort may be a concern for intranasal acute seizure therapies due to previous experience with use of atomized IV formulations or prior investigational nasal formulations. For example, in a study evaluating intranasal use of an injectable midazolam solution (pH of ∼ 3) when used by this route to abort seizures, patients reported considerable pain immediately after administration [5], [23]. An intranasal therapy using diazepam with an organic solvent was previously discontinued due to suboptimal absorption and local adverse events that were felt to be potentially related to the solvent [5].

#### Current evidence

The assumption that mucosal irritation should be expected with intranasal administration of medication stems from use of formulations not approved by the U.S. FDA for this use. Recently available FDA-approved intranasal formulations of acute seizure rescue medications were specifically designed and developed to reduce the potential for nasal irritation [18]. The pH range of the commercially available FDA-approved intranasal midazolam formulation (Nayzilam®; UCB, Smyrna, GA) is approximately 5 to 9 [16]. Solvents used in the intranasal formulations also differ, and thus have differing profiles relative to producing nasal irritation [15], [16]. The FDA-approved diazepam nasal spray (Valtoco®; Neurelis, San Diego, CA) is a vitamin E–based solution (the formulation components diazepam and tocopherol have neutral pH) that enhances the solubility of diazepam without the toxicities encountered when organic vehicles are used [18], [24], [25], [26].

#### Conclusion

Substantial mucosal nasal irritation should NOT be expected with intermittent use of acute intranasal seizure therapies.

### Misconception? Concomitant use of benzodiazepines impacts the safety profile of intermittent use of benzodiazepine acute seizure therapies

#### Background

Benzodiazepines as ASMs have an important role in epilepsy management. The successful use of chronic benzodiazepines, such as clobazam in patients with Lennox-Gastaut syndrome [27], depends on the specific medication. Despite maintenance treatment, seizure emergencies may still occur, which prompt treatment with a benzodiazepine acute seizure therapy [1], [2]. Because benzodiazepines have been associated with IV usage- and dose-related cardiorespiratory effects during treatment for status epilepticus [12], [22], patients using concomitant maintenance benzodiazepines were excluded from studies involving intranasal use of midazolam [20], [21].

#### Current evidence

A phase 3 study of diazepam nasal spray permitted patient use of benzodiazepines as chronic maintenance therapy [18]. A secondary analysis assessed the safety profile of diazepam nasal spray in patients treated with concomitant benzodiazepines, primarily oral formulations [18]. No cardiorespiratory events or serious treatment-related adverse events were observed in patients taking concomitant benzodiazepines or in the rest of the patient population. The safety profile for patients taking concomitant benzodiazepines was generally similar to other subgroups of patients in the study (e.g., those grouped by age, monthly usage of diazepam nasal spray, and seasonal allergy or rhinitis). In addition, the overall safety profile of diazepam nasal spray was consistent with the reported safety profile of rectal diazepam [18].

#### Conclusion

Concomitant use of chronic benzodiazepines does NOT appear to have an impact on the safety profile of intermittent use of diazepam nasal spray for acute seizure therapy (no published data for intranasal midazolam).

## Patient populations

These concepts are associated with age groups of patients experiencing seizure emergencies.

### Misconception? Acute seizure therapy is intermittently necessary for acute seizure control in children with epilepsy but is not necessary for adult PWE

#### Background

Acute seizure therapy is prescribed at a different rate for children and adults with epilepsy. One cross-sectional observational study surveyed 100 families that included a child with epilepsy aged ≤ 21 years [28]. A questionnaire focused on the use of acute seizure therapies was utilized to survey family members and this information was corroborated by medical chart review. Eighty-seven percent of the families reported that patients were prescribed an acute seizure rescue therapy [28]. In contrast, a study of medical records in the Columbia/Yale Antiepileptic Drug Database found that of 612 adult patients with epilepsy and seizure clusters (aged ≥ 16 years), 44% were prescribed acute seizure rescue therapy [29]. Reasons for a substantial difference in age-related studies includes different cohorts, year of study, method of study design, and formulation of the acute rescue therapy evaluated [29]. Additionally, pediatric patients are likely to have a parental caregiver who is willing and able to administer rescue medication while an adult patient might not.

#### Current evidence

The prevalence of epilepsy is 2.6 to 6 million people in Europe and >3 million in the United States [30], [31]. Approximately 30% to 40% of all patients with epilepsy have drug-resistant seizures, continuing to have seizures despite chronic use of maintenance ASM [1], [32]. Patients with drug-resistant epilepsy of any age are at higher risk for seizure emergencies [33]. Among patients with drug-resistant epilepsy, there are both adults [29] and children with seizure clusters [1].

Untreated seizure emergencies can have devastating consequences. Therefore, treatment in the form of an acute ASM is needed to interrupt seizures and prevent progression to acute repetitive seizures or status epilepticus that would require emergency care utilizing accessible healthcare resources and facilities [2], [5]. Because of the risk to suffer negative outcomes, any patient with a potential for seizure urgencies or emergencies should be considered for acute seizure therapy [2].

#### Conclusion

Intermittent use of acute seizure therapy is needed NOT ONLY for children with epilepsy, but also for adults.

### Misconception? Written seizure action plans (SAPs) and acute seizure action plans (ASAPs) only apply to school-aged children with seizures

#### Background

Written treatment plans are often a requirement for children with epilepsy who attend public schools in case medication needs to be administered by school personnel [34], [35]. In contrast, written SAPs are underutilized in adults [36]. This is due to the belief that adults are generally able to take care of themselves and, therefore, an SAP would not be used if available [37]. Thus, SAPs may not be included during transitioning care of a patient with epilepsy from pediatrics to adult healthcare settings [36]. In addition, unlike school requirements, work settings that employ adults with epilepsy frequently neither require SAPs nor have a nurse on site. Also, although a parent is required to disclose their child’s medical condition, an adult may live in fear of the epilepsy-related stigma they have experienced and be less willing to disclose their condition at work that provides colleagues with an SAP [36], [38]. The assumption that adults will remember and share their healthcare professional’s recommendations with others coupled with a short duration of visit (which might be especially packed if visits are infrequent) is often suboptimal to result in a comprehensive SAP [36].

#### Current evidence

For other chronic medical conditions that require emergency treatment (e.g., diabetes, asthma, chronic obstructive pulmonary disease [COPD]), self-management, education, and action plans demonstrate that written plans are beneficial for patients of all ages [39], [40], [41]. In these cases, action plans may result in better disease control, reduced healthcare utilization, and improvement in patient quality of life. For example, a study of 363 patients with (median age 23 years) found that an action helped improve the patient’s initial knowledge of their condition, provided greater confidence regarding asthma control, and improved their quality of life [40]. Additionally, a Cochrane review of studies that included adult patients with COPD found that use of an action plan targeting exacerbations and having a short educational component combined with ongoing support resulted in reduced healthcare utilization and better understanding of treatment [41].

Seizure action plans for children with epilepsy have shown benefits for patients and caregivers. One study looked at the effects of a new standardized SAP form in a pediatric neurology clinic and found that SAPs increased parental knowledge and confidence about their child’s epilepsy treatment compared with those who did not receive a plan [42]. Understanding a treatment plan and communication with physicians also important for adult patients [36]. Therefore, it is important for physicians to consider customized written SAPs for a patient of any age [36].

Guidelines and templates for SAPs allow standardization [38]. Forms can be customized to an individual and promote an understanding of their treatment. Templates can be made available in electronic medical records systems, allowing for improved access and the ability to individualize plans for each patient. Brief and concise SAPs can foster communication with healthcare professionals, as well as the patient’s family, friends, and coworkers [38]. Similar to asthma plans for children and for adults, prompt identification and appropriate treatment are needed in seizure emergencies [36], [43]. Early administration of an intranasal rescue therapy for acute seizure management may be enhanced by an acute SAP (ASAP) to facilitate rapid and effective care during a seizure emergency [4], [38]. An expert panel recommended ASAPs for all adults with epilepsy and specifically those with new-onset epilepsy or with ongoing convulsive seizures who had > 1 seizure in the past year [38].

#### Conclusion

Written SAPs are useful NOT ONLY for school-aged children, BUT ALSO for adults with epilepsy.

## Drug formulations

These concepts are associated with comparisons among medications and appropriate usage of different formulations for acute seizure therapies.

### Misconception? When administered intranasally as a spray, all benzodiazepine formulations work the same way

#### Background

The limitations of IV and rectal administration of ASMs for seizure emergencies led to investigation of potential fast-acting intranasal therapy that could be easily administered by nonmedical individuals [23]. Considerations for selecting an intranasal acute seizure rescue therapy include patient tolerability, small volume of solution, and the capacity for rapid administration [23]. Atomized benzodiazepine solutions were used in many institutions before the FDA approval of intranasal formulations [2]. In some cases, there is a false belief that atomized benzodiazepine solutions using a nasal or rectal route of administration is equivalent to other FDA approved products currently available as intranasal acute seizure rescue therapies.

#### Current evidence

Potential shortcomings of intranasal administration of solutions designed for IV use include inadvertent swallowing of medication, nasal irritation, and variable absorption and bioavailability (absolute levels and time to reach therapeutic levels) [5], [8]. Injectables given via an atomizer may require large volumes to deliver adequate doses of medication and exceed the capacity of the nasal cavity (>200 µL) for absorption and therefore may be associated with swallowing or leakage of medication from the nose [5], [20]. For example, IV solutions of midazolam used intranasally require a large volume (e.g., 1 mL) [5]. Furthermore, atomized IV formulations may also be associated with nasal irritation because of a low pH of the drug more appropriate for injection [5].

These limitations are less likely to be associated with formulations that are specifically designed for intranasal administration [5], [18], [20]. Nasal-specific ASM formulations are designed to provide doses less than 200 µL to deliver medication in the appropriate concentration to that optimizes absorption [5]. These formulations are specifically designed for nasal administration, incorporating small volumes of drug and a neutral pH appropriate for use. In addition, solvents used to facilitate absorption differ by formulation with organic solvents or vitamin E possessing differences in local safety and tolerability [5], [15], [16].

#### Conclusion

When used as an intranasal rescue therapy, all benzodiazepine formulations DO NOT work the same way.

### Misconception? Oral benzodiazepines have shortcomings as acute seizure therapy in adults

#### Background

Oral ASMs (e.g., clonazepam, diazepam, or lorazepam tablets and wafer formulations [oral disintegrating tablets]) have been used predominantly in adult patients as a common route of administration and are more socially acceptable than rectal administration [3], [4]. In a database review of medical records for adult patients with epilepsy and seizure clusters (n = 612), the most commonly prescribed rescue medication was oral lorazepam (28.9% of patients), followed by rectal diazepam (7.8%) [29] before the availability of intranasal acute seizure therapies.

#### Current evidence

The potential shortcomings of orally administrated tablets and oral formulations include aspiration risk, delayed time to efficacy, first-pass metabolism, and incomplete ingestion limited by swallowing [8], [44], [45]. Furthermore, delayed absorption may occur when taken with food [46]. Mechanical limitations also may exist during seizure emergencies when trying to open the patient’s lower jaw to administer treatment, resulting in difficult and unsafe practice [47]. As a consequence, the time to seizure cessation for oral medications may be longer than an hour, making this route inadequate for treatment of seizure urgencies and emergencies and reducing the likelihood of a favorable response [4].

Intranasal formulations of acute rescue medication provide advantages over limitations imposed by rectal administration and the ability to overcome those associated with oral agents [4], [18]. Intranasal delivery devices are portable, easily accessible, readily accepted, and simple to use during a seizure cluster [48]. Nasal formulations are absorbed by the mucosa and bypass the gastrointestinal first-pass effect [18]. In addition, there is rapid bioavailability and shorter time to clinical efficacy [4], [49]. Further, nasal administration does not require significant patient cooperation or have mechanical challenges of administration, including positioning or risk of injury, such as during an attempt at oral administration during a seizure.

#### Conclusion

Oral benzodiazepines DO have shortcomings as an acute seizure therapy in adults.

## Administration

These concepts are associated with who can administer acute seizure therapy and when these therapies should be administered.

### Misconception? Acute seizure therapy can be safely and easily administered by nonmedical individuals such as care partners and school personnel and in some cases may be self-administered by the patient

#### Background

Intravenous administration is the gold standard therapy for aborting seizure emergencies including status epilepticus [4]. The time to onset of action for IV lorazepam is approximately 1 to 3 min [4]. The IV route is unsafe in untrained hands and out-of-hospital settings and requires administration by medical professionals in a controlled environment [4], [5]. Similarly, intramuscular (IM) administration requires an established skill set for safe administration and is best administered by trained healthcare professionals [5].

#### Current evidence

The time and access required to secure IV administration contributes to the need for developing an intermittent acute seizure rescue therapy for use outside the hospital setting [4]. IV administration is optimal in hospital settings and is not suitable for at-home treatment of seizure emergencies [3]. In a study of prehospital treatment of status epilepticus, IM midazolam was shown to be at least as safe and effective as comparable doses of IV lorazepam when medication was administered by experienced paramedics [50]. When healthcare personnel are unavailable, portable products are essential that can be used easily by nonmedical caregivers, and when seizures do not impede awareness, potentially by the patients themselves [5], [48].

Acute seizure rescue therapies for seizure clusters that occur in the home or community setting should be easy to use by lay personnel, including caregivers [48]. Safe use has been deployed by caregivers, including babysitters and other nonmedical individuals [2], [5], [38]. In the community setting, caregivers found diazepam nasal spray easy to use [48], and doses were successfully given in 99% of treatments [18]. Caregivers administering intranasal midazolam reported reduced anxiety and increased confidence to travel [51].

PWE in a phase 3 open-label safety study of diazepam nasal spray and their caregivers completed survey questionnaires about usage of the study therapy; 27 of the 163 patients who used intranasal diazepam spray reported self-administering medication [48]. Of all doses delivered in the study, 26.7% were administered in these patients, with a low rate of errors (1.1%) during delivery [48]. Of the 27 patients, 21 (77.8%) responded that self-administration was either extremely easy (n = 11) or very easy (n = 10) [48].

#### Conclusion

Non-parenteral formulations of acute seizure rescue therapy, including rectal gels and nasal sprays, CAN be administered by nonmedical individuals such as care partners and school personnel and in some cases self-administered by patients themselves.

### Misconception? In patients having a seizure emergency, always wait 5 min or until a second seizure occurs before administering an acute seizure therapy

#### Background

In 2016, the Guideline Committee of the American Epilepsy Society provided guidelines for the treatment of convulsive status epilepticus in children and adult patients with epilepsy [22]. Based on 4 class 1 studies, 2 class 2 studies, and 32 class 3 studies, these guidelines specifically designated 5 min as the operational definition for *status epilepticus.* Because the majority of seizures are brief, if seizures last longer than 5 min they are likely to continue. Therefore, benzodiazepine treatment for convulsive status epilepticus is begun at 5 min to minimize risks of seizure prolongation and the consequences of overtreatment [22].

#### Current evidence

Acute repetitive seizures, like status epilepticus, are a medical emergency [52]. They both require diagnosis and treatment as soon as possible to obtain the best response [6]. Benzodiazepines are the first line of therapy [4]. However, the use of benzodiazepines for acute seizure emergencies may be subject to delay, underdosing, or omitted entirely [53]. Delayed treatment with a benzodiazepine has been associated with longer times to achieve seizure control, lower effectiveness, more adverse events, and worse outcomes [6], [53]. A delay of > 30 min to initiate treatment for status epilepticus was shown to be associated with longer seizure durations, benzodiazepine resistance, and long-term consequences, such as irreversible neuronal injury and functional deficits [54], [55], [56]. In drug-resistant epilepsy, long-term sequelae including brain atrophy and progressive features such as cognitive decline have been observed [57], [58].

Two specific situations arise that deserve special consideration. First, patients with a history of a prolonged seizures or status epilepticus are at higher risk to have a subsequent future episodes of prolonged seizures and status epilepticus [59]. Second, use of an acute seizure therapy is most effective when initiated early in the seizure cluster [6], [60]. Waiting for a second seizure to occur is unnecessary when the first seizure is characteristic of a patient’s seizure cluster in those with an established pattern of recurrence. When to intervene during a seizure cluster, such as waiting for a second seizure or outlining specific scenarios for treatment, is not specified or limited by prescribing information of medications used for acute rescue therapy [14], [15], [16]. Seizure clusters are usually easily recognized by a close family member, friend, or care partner.

Data obtained from patient diaries in a phase 3, open-label, repeat-dose safety study of diazepam nasal spray was evaluated in a post hoc analysis to examine the time to administer medication and the time until seizures ceased [60]. This analysis evaluated the impact of a single dose of diazepam nasal spray on seizure duration. Rapid administration was feasible with a median time of 2 min from seizure onset and the median time from administration to cessation of seizures was 4 min [60].

#### Conclusion

For patients with a history of prolonged seizures and for those who experience recurrent seizure clusters, waiting 5 min or until a second seizure occurs to initiate treatment is NOT appropriate for patients with an established pattern.

## Effectiveness

These concepts are associated with clinical results based on the route of administration and use of pharmacokinetic characteristics for assessing the effectiveness of acute seizure therapies.

### Misconception? Clinical endpoints are the same irrespective of the route of administration for patients requiring an acute seizure rescue therapy

#### Background

Oral, rectal, and intranasal formulations for acute seizure rescue therapy allow for a variety of routes of administration.

#### Current evidence

The rate of absorption for medication used for intermittent acute seizure rescue therapy varies based on pharmacologic and pharmacodynamic factors as well as pharmacokinetics including the route of administration [5]. As an example, buccal administration of medicine may inadvertently be partially swallowed, and rectal administration has challenges that include mechanical limitations that involve positioning the patient [5], [6] Further, rectal diazepam has greater interpatient variation in bioavailability compared with the intranasal route [25]. Also, there may be unanticipated differences in bioavailability, safety, and efficacy when the same drug formulation is administered through different routes. For example, IV midazolam has a rapid time to maximum plasma concentration (T_max_) and to clinical seizure cessation [4], but poor tolerability and leakage of medication may arise, potentially leading to ineffective dosing when this formulation is used via the intranasal route [5]. Under ideal conditions with a 10-hour fast before administration, oral diazepam has been shown to have a modestly faster mean T_max_ than intranasal and rectal diazepam; however, use of oral diazepam with food would be expected to result in greater pharmacokinetic variability [25].

Use of intranasal formulations may reduce time-to-treatment delays in drug delivery [4]. The time to seizure cessation also varies by route of administration. The intranasal route is the most rapid delivery route outside of the hospital setting [4]. There is a comparable time to seizure cessation for IV and intranasal administration when including the time needed to establish an IV line [4]. For diazepam, although no clinical head-to-head studies have been done, the FDA notes the route of administration involving an intranasal spray is clinically superior to rectal administration due to more favorable ease of use, which may allow for easier administration of medication during a seizure [61].

#### Conclusion

Clinical endpoints results are NOT the same irrespective of the route of administration for an acute seizure rescue therapy.

### Misconception? The time to effectiveness of an acute seizure rescue therapy can be determined using pharmacokinetic characteristics

#### Background

There is a belief that the T_max_ of a medication is equivalent to the onset of action for its use as intermittent acute seizure rescue therapy.

#### Current evidence

In the recent literature, the time to effectiveness for medications that are used for acute seizure urgencies and emergencies was not shown to be directly proportional to the T_max_ [4], [6], [53], [60]. Instead, the time to onset of effectiveness has been linked to when plasma concentrations reach a therapeutic threshold [62]. This may be substantially different from the T_max_ and instead was shown in an animal model to become effective with a minimal steady-state plasma level of medication to cause seizure threshold elevation to function as an acute antiseizure treatment [62]. In one study, ictal/*peri*-ictal and interictal diazepam plasma concentrations in the first 6 h following intranasal administration were sustained above the minimal concentration shown to elevate the seizure threshold [63].

#### Conclusion

The time to an acute seizure rescue therapy’s effectiveness CANNOT be determined using pharmacokinetic characteristics.

### Misconception? Seasonal allergies and rhinitis do not appear to have an adverse clinical impact on effectiveness of intranasal drug delivery

#### Background

Seasonal allergies may cause inflammation that has the potential to tighten airway mucosa and reduce nasal absorption of topically deposited molecules of medication [64]. However, the effects of seasonal allergies on intranasal formulations have been mild to moderate but lack clinical impactfulness [65]. In a study of patients treated with intranasal triamcinolone acetonide, pharmacokinetic parameters were similar between patients with and without nasal inflammation from allergic rhinitis [66]. In another study evaluating zolmitriptan nasal spray for migraine, efficacy was not affected by the presence of rhinitis [67].

#### Current evidence

For intranasal midazolam, patients with allergic rhinitis have not yet been formally evaluated [20]. In a diazepam nasal spray phase 3 safety study, patients with seasonal allergies or rhinitis were permitted to enroll, but the number of nasal sprays administered per seizure cluster was used as a proxy for effectiveness [18], [65]. With this criterion, no impact on the proportion of seizure clusters that required a second dose of medication was seen in patients with and without seasonal allergies or rhinitis (11% vs 8%, respectively). These results support effectiveness for the first dose of the intranasal medication in the vast majority of patients with seasonal allergies or rhinitis.

#### Conclusion

Seasonal allergies and rhinitis do NOT appear to have a clinical impact on the effectiveness of intermittent intranasal drug delivery for acute seizure termination.

## Conclusions

Current priorities for treating patients with epilepsy should include an up-to-date SAP. Plans for patients who experience seizure urgencies and emergencies should include directions for intermittent use of acute seizure rescue therapies. An array of therapeutic options are currently available, to provide acute seizure rescue treatment for a wide range of patients with epilepsy. Clinicians should be aware of how to distinguish the practical benefits and limitations of each formulation and route of administration so that using acute seizure rescue therapies maximizes effectiveness and safety. This necessitates understanding misconceptions associated with intermittent use of acute seizure rescue therapies to provide the best practices in epilepsy management.

**Dr Tatum** received a stipend from Elsevier as immediate past Editor-in-Chief of *Epilepsy & Behavior Reports* and is on the Editorial Board of *Journal of Clinical Neurophysiology*. He has received grant funding from Eisai Inc., Martin Family Foundation, Mayo Clinic, and McElvey Fund, and industry research support from Cerevel, Engage, LivaNova, UCB and Xenon. He has served as a consultant for Bioserenity; Neurelis, Inc.; Natus and Zimmer-Biomet. He has received royalties from Demos, Springer Publishers, and Wiley. He has patents/patents pending on intraoperative monitoring devices (#62527896; #62770362). **Dr Glauser** is a consultant for Clarigent Health and Neurelis, Inc. He receives research support from the National Institutes of Health. **Dr Peters** has served as a speaker and consultant for Neurelis, Inc.; SK Life Science; and Jazz Pharmaceuticals. **Dr Verma** is a consultant and member of the speakers bureau for Aquestive Therapeutics; Neurelis, Inc; SK Pharmaceuticals, and UCB. **Dr Weatherspoon** is a consultant and speaker for Neurelis, Inc. **Dr Benbadis** is a consultant or member of an advisory board for Eisai Inc.; Jazz Pharmaceuticals; Neurelis, Inc; SK Life Science; Sunovion; Takeda; and UCB. He is a member of the speakers bureau for Aquestive; Eisai Inc.; Jazz Pharmaceuticals; SK Life Science; Sunovion; and UCB. **Dr Becker** is a consultant/speaker for Neurelis, Inc; SK Life Science; Supernus Pharmaceuticals; and UCB; is a speaker for Jazz Pharmaceuticals; and received research support from SK Life Science. **Dr Puri** is a consultant for Eisai Inc. and a speaker and consultant for Neurelis, Inc. **Dr Smith** is a consultant for Azurity Pharmaceuticals and a consultant and speaker for Neurelis, Inc., and SK Life Science. **Dr Misra** and **Dr Rabinowicz** are employees of and have received stock options from Neurelis, Inc. **Dr Carrazana** is an employee of and has received stock and stock options from Neurelis, Inc.

## Declaration of Competing Interest

The authors declare that they have no known competing financial interests or personal relationships that could have appeared to influence the work reported in this paper.
